# A digital intervention targeting cognitive control network dysfunction in middle age and older adults with major depression

**DOI:** 10.1038/s41398-021-01386-8

**Published:** 2021-05-04

**Authors:** Faith M. Gunning, Joaquin A. Anguera, Lindsay W. Victoria, Patricia A. Areán

**Affiliations:** 1 Department of Psychiatry, Weill Cornell Medicine, New York, NY USA; 2 Departments of Neurology and Psychiatry, University of California San Francisco, San Francisco, CA USA; 3 Department of Psychiatry and Behavioral Sciences, University of Washington, Seattle, WA USA

**Keywords:** Neuroscience, Depression

## Abstract

Nonpharmacological interventions targeting putative network mechanisms of major depressive disorder (MDD) may represent novel treatments. This mechanistic study investigates how a video game-like intervention, designed to improve cognitive control network (CCN) functioning by targeting multitasking, influences the CCN of middle-aged and older adults with MDD. The sample consisted of 34 adults aged 45–75 with SCID-defined diagnosis of MDD, Hamilton depression rating scale scores ≥20, and a deficit in cognitive control. Participants were instructed to play at home for 20–25 min per day, at least 5 times per week, for 4 weeks. Evidence of target engagement was defined a priori as >2/3 of participants showing CCN improvement. CCN engagement was defined as a change in a Z score of ≥0.5 on functional magnetic resonance imaging (fMRI) in activation and functional connectivity of the CCN during task-based and resting-state fMRI, respectively. 74% of participants showed a change in activation of the CCN, and 72% showed an increase in resting-state functional connectivity. Sixty-eight percent demonstrated improved cognitive control function, measured as either improvement on sustained attention or working memory performance or reduced self-reported symptoms of apathy on the frontal systems behavioral scale (FrsBe). Participants also reported a significant reduction in mood symptoms measured by PHQ-9. A remotely deployed neuroscience-informed video game-like intervention improves both CCN functions and mood in middle-aged and older adults with MDD. This easily-disseminated intervention may rescue CCN dysfunction present in a substantial subset of middle-aged and older adults with MDD.

## Introduction

At least one-third of individuals treated with antidepressant medications or psychotherapy do not achieve adequate response, even after multiple treatments trials^1–3^. Even when standard treatments improve mood symptoms, many individuals, especially older adults, are left with persistent executive dysfunction^4–7^ that is associated with disability^5–7^, and increased risk of depression recurrence^5–9^. Thus far, alternative pharmacologic approaches designed to target comorbid executive dysfunction demonstrate limited efficacy^8,10^. Likewise, psychotherapy we optimized to treat individuals suffering from major depressive disorder (MDD) with executive dysfunction improved mood symptoms^11,12^, but not executive dysfunction^13^. Novel strategies are needed that target persistent dysfunction among individuals with MDD.

Executive dysfunction present in older individuals with MDD appears to reflect inefficient functioning of the cognitive control network (CCN)^14–18^. The CCN, comprised of the dorsal anterior cingulate cortex (dACC), dorsolateral prefrontal cortices, and posterior parietal cortices support the flexible maintenance of goal-directed behavior in the face of changing internal and environmental demands^19,20^. Nonpharmacological interventions that aim to rescue CCN network dysfunctions could therefore represent a promising, personalized approach for the large subset of individuals with MDD who have an inefficient CCN and are often less likely to benefit from standard treatments.

One method to target CCN dysfunction applies digital cognitive interventions designed to improve CCN functions through the repetitive engagement of the network. We conducted a preliminary, proof of concept RCT of a video game-like intervention designed to target the CCN by improving age-related deficits in multitasking^13^. In that study, individuals over the age of 60 in a major depressive episode were randomized to problem-solving therapy (PST), efficacious psychotherapy for older adults with depression, or digital intervention. Although the two groups showed a similar improvement in depression, only the group randomized to the multitasking game-like condition showed significant improvement in working memory and sustained attention performance^13^.

The results of this pilot RCT shed limited light on the mechanism of action of the intervention. In fact, nearly all non-pharmacologic intervention studies in MDD focus on demonstrating efficacy, without confirming target engagement^21^. That is they may show clinical benefit without demonstrating how an intervention engages the hypothesized target. In the present study, we aimed to determine whether an algorithmically delivered video game-like intervention targeting multitasking can modulate CCN function in middle-aged and older adults with MDD. We hypothesized that after 4 weeks of training with this intervention at least 2/3 of participants would show a significant improvement in focal activation of the anterior CCN and an increase in functional connectivity of the CCN at rest. In addition, we assessed whether improvement in CCN functioning would be reflected in a similar degree and frequency of improvement demonstrated on either measure of cognitive control performance as assessed by a visual continuous performance task (CPT) and a delayed recognition working memory task or decreased complaints on the apathy subscale of the frontal systems behavior scale (FrSBe)^22^.

## Materials and methods

### Study design

This study was the proof-of-concept phase of a two-phase clinical trial to evaluate mechanisms of action of the video game-like intervention, specifically engagement of CCN as measured by functional magnetic resonance imaging (fMRI), computerized sustained attention, and working memory tasks, and self-reported symptoms of apathy. The study used a single-arm, open-label design. Measures of CCN target engagement were administered at baseline/pre-intervention and week 4/post-intervention with analysis that focused on changes in CCN function over 4 weeks.

### Participants

Participants were adults aged 45–75. Prior to study entry, MDD diagnosis was confirmed by Structured Clinical Interview for DSM-V (SCID)^23^, conducted by a study clinician. Eligibility required baseline Hamilton depression rating scale^24^ scores ≥20 (i.e., moderate severity). Participants were required to demonstrate a deficit in cognitive control, defined as >1 standard deviation (SD) below the mean for their age and education on at least one of the following: Stroop Color Word Interference^25^, Trail Making Part B^26^, or FrSBe^22^. Participants were either not currently on antidepressants or on a stable dose for at least 12 weeks, with no intent to change dose during study participation. Individuals were excluded if they had a mini-mental state examination (MMSE)^27^ score >1 SD below the mean for their age and education level, or a contraindication to MRI. Individuals with a history of neurologic illnesses, psychosis or mania, or acute medical illnesses were also excluded.

All participants provided written informed consent, as per the study protocol approved by the IRBs of Weill Cornell Medicine, University of Washington, and the Nathan Kline Institute.

### Intervention

The intervention is a self-guided therapeutic video game-like intervention (Project: Evolution [EVO], Akili Interactive Labs, Cambridge, MA) that involves a combination of visuomotor and perceptual discrimination tasks intended to adaptively treat multi-tasking deficiencies^28^. The game begins with an assessment of the participants’ multi-tasking cost, i.e., tests of reaction time alone, navigation alone, and then the two domains combined. This sets the level of difficulty for each participant, personalizing the training experience. Improvement throughout the intervention transports the participant to different visual “worlds”, designed to immerse the player and enhance the depth of engagement and compliance. Frequent performance assessments are embedded in the program to determine the level of improvement during training and adaptively set a therapeutic regimen based on the participant’s performance. Thus, the participant is challenged to continue to improve upon their own performance to reach the next level. The same adaptive mechanics utilized in the assessment are employed in the training sessions which keep the player at ~80% accuracy.

The intervention relied on at-home use of the therapeutic video game, delivered via Apple iPad, and 4 weekly in-person visits with a Masters or PhD-level care manager who provided instruction and technical support for participation in the intervention. At each study visit the participants completed the patient health questionnaire-9 (PHQ-9)^29^ to assess depressive symptoms as part of safety and mood monitoring.

During the first session, care managers introduced the game, observed participants’ completion of a practice session, and set an action plan with participants to ensure regular gameplay. During the weekly follow-up sessions, care managers reviewed the adherence and action plan and a dashboard within the application tracking performance and time played. They worked with participants to adjust the action plan as needed if adherence was low, defined as less than 5 days of gameplay.

The intervention required 20–25 min of gameplay for a minimum of 5 days and up to 7 days per week. The game locked after five sessions per day.

### Measures of target engagement

The MRI, computerized cognitive control tasks, and self-report measures of CCN function were collected at baseline and following 4 weeks of the intervention.

#### Circuitry

CCN circuitry was measured by assessing focal activation of the anterior CCN using task-based fMRI during a Stroop/Flanker task and resting-state functional connectivity (rsFC) of the CCN. Details related to scanning parameters, image acquisition, and preprocessing steps are described in the Supplementary Information.

#### Stroop/flanker task

Participants viewed three color words on the screen: the central congruent or incongruent Stroop^25^ target word and two surrounding Flanker^30^ words. They responded to the ink color of the central target word with a button press while ignoring multiple sources of cognitive interference: what the word says and the surrounding distractor word. The main contrast of interest for the task-based MRI analyses focused on the congruency of the Stroop targets: congruent (e.g., “purple” in purple ink) vs. incongruent (e.g., “purple” in orange ink). See Supplementary Fig. 1 for more details.

Following preprocessing of the task-based data, we extracted percent signal activation during the Stroop/Flanker task in regions of the anterior portion of the CCN: middle frontal gyrus (MFG) and dACC from baseline to week 4 MRI. Beta values were extracted for incongruent trials only since they had the highest cognitive control load. Our z-scored measure of target engagement for task-based activation was calculated as follows: (week 4 incongruent MFG and dACC activation − baseline MFG and dACC activation)/baseline SD of MFG and dACC activation. The mean and SD of the extracted beta values were calculated within-region.

#### rsFC

We conducted a seed-based rsFC analysis to investigate the change in connectivity among nodes of the anterior portion of the CCN (i.e., MFG and dACC) following 4 weeks of the intervention.

To examine target engagement, we measured within-CCN connectivity between MFG and dACC. Following preprocessing of the resting-state data, we extracted correlation coefficients between these regions at baseline and week 4 of EVO with 4 mm spherical seeds placed bilaterally in the MFG (MNI coordinates −36/36, 28, 34) and the dACC (MNI coordinates −4/4, 30, 22). Our z-scored measure of target engagement for the rsFC analysis was calculated as follows: (week 4 partial correlation between MFG and dACC − baseline partial correlation between MFG and dACC)/baseline SD of partial correlation between MFG and dACC.

#### Cognitive control performance

Two cognitive tasks were used to measure target engagement of the CCN: (1) a delayed recognition working memory task (called “AID” for attending or ignore distractor)^31^; (2) a continuous performance test (CPT)^32^, a measure of sustained attention.

#### AID

The AID includes aspects of visual information processing that are held constant (“Is the final face shown the same as the first?”) while other task demands are manipulated. Our analysis focused on the “ignore distractor” (ID) condition, during which participants are instructed to ignore the distracting face while maintaining the representation of the cue face. We analyzed RT during this condition^13,28^ and calculated Z-scores as follows: (week 4 ignore distractor RT − baseline ignore distractor RT)/baseline SD of ignoring distractor RT.

#### CPT

We used a CPT^32^, a 23-min, fixed interval, visual CPT that we modeled after the test of variable attention (TOVA)^33^. Participants were instructed to respond to the target (white square at the top edge of the screen) and to ignore the non-target (white square at the bottom edge of the screen). We analyzed response time in the sustained attention condition (target stimuli appears in 22% of trails) and calculated *Z*-scores as follows: (week 4 sustained attention RT − baseline sustained attention RT)/baseline SD of sustained attention RT.

#### Self-reported cognitive control

We used the apathy subscale of the FrSBe^22^, a self-report scale that measures executive function. We examined the apathy subscale to capture cognitive symptoms that are likely not represented in the other cognitive measures. We analyzed the FrSBe apathy subscale and calculated z-scores as follows: (week 4 score − baseline score)/baseline SD of the score.

### Data analysis and go/no-go decision

The primary purpose of this phase of the clinical trial was to establish whether or not the intervention engaged the hypothesized CCN target. We defined the go/no-go criteria a priori, informed by other proof-of-concept trial methodology^34,35^. We determined that 66% of the sample would need to show significant improvement in the CCN on fMRI (task-based activation and rsFC) and either CCN task performance or self-reported symptoms of cognitive control. Significant improvement was defined as Z-scores ≥ 0.5 for pre- to post-EVO change in CCN task-based activation and functional connectivity, as well as CPT and/or AID performance, or FrSBe self-report score.

Secondary analyses of CCN circuitry included exploratory whole-brain analyses of Stroop/Flanker task performance pre- and post-EVO, as well as whole-brain seed-based exploration of changes in rsFC from pre- to post-EVO. The methodological details of these analyses are described in Supplementary Information. We also explored the association between changes in rsFC and post-intervention performance and self-reported CCN function with correlational analyses comparing the Z-scored change in rsFC pre- to post-EVO to post-EVO performance on the CPT and AID tasks and post-EVO self-reported FrSBe scores. These results are reported as Pearson’s correlation coefficients.

Exploratory analysis of depression outcomes consisted of repeated measures ANOVA to determine changes in mood over time, the proportion of participants exhibiting response (defined as a 50% decrease in PHQ-9 scores from baseline to 4 weeks), and remission (defined as a PHQ-9 score of less than 5 at 4 weeks).

## Results

### Sample characteristics

Forty-one individuals were enrolled in the trial. Seven participants were excluded from the analysis (see Supplementary Fig. 2 for participant flow diagram), yielding a final sample of 34 participants. Seventy-one percent (*N* = 24) of the sample was female, aged 45–75 years (*M* = 61.6 years, SD = 9.4). The mean education level was 15.8 years (SD = 2.2) and the mean MMSE score was 28.9 (SD = 1.0). At the baseline visit, the mean HAM-D score was 24.4 (SD = 4.9). All subjects completed 4 weeks of the intervention.

### Target engagement

#### CCN circuity (task-based activation)

**Fig. 1:**
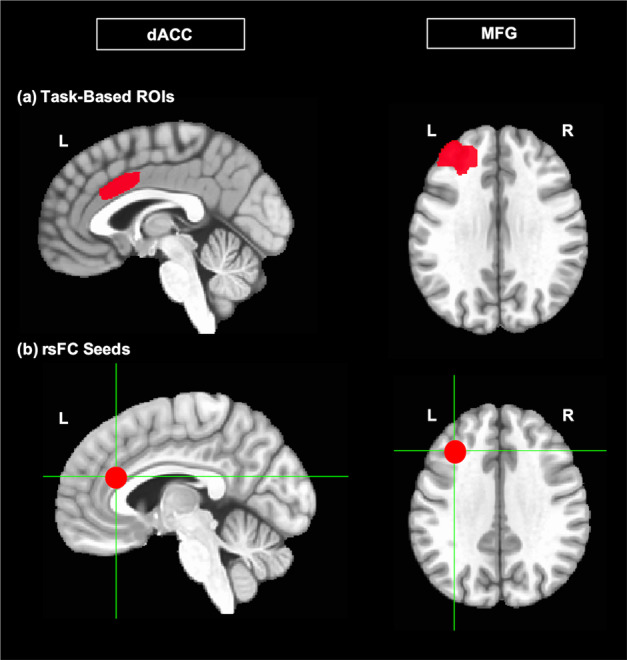
A priori regions of interest (ROIs) in the cognitive control network (CCN) defined for circuitry-based analysis: (left) middle frontal gyrus (MFG); (right) dorsal anterior cingulate cortex (dACC). **a** ROIs from which beta values were extracted for Stroop/Flanker task-based analysis of target engagement. **b** Placement of 4 mm spherical seeds in the MFG and dACC for resting-state functional connectivity (rsFC) analysis of target engagement.

To assess activation, beta values were extracted from AFNI Daemon atlas-based ROIs in the MFG and dACC (Fig. 1a). Twenty-one of the 34 participants (74%) showed greater change in activation of the CCN (MFG and/or dACC) for incongruent trials (i.e., trials with a higher cognitive control load) after 4 weeks of EVO (Table 1).

**Table 1:** Proportion of participants meeting target engagement goal for each level of analysis of cognitive control function.

Level of analysis	*N* (%)
Cognitive control performance: AID/CPT	23/34 (68%)
Self-reported cognitive control symptoms: FrSBe	23/34 (68%)
CCN circuitry (task-based activation)	25/34 (74%)
CCN circuitry (rsFC)	21/29 (72%)

*CPT* continuous performance task, *FrSBe* frontal systems behavior scale, *CCN* cognitive control network, *rsFC* resting-state functional connectivity.

The successful target engagement goal was defined as 66% of participants with significant improvement (z-score ≥ 0.5) in CCN measures (performance, self-report, circuitry) following 4 weeks of EVO.

#### CCN circuitry (rsFC)

Five participants were excluded from the rsFC analysis due to a high degree of motion in the resting-state scan (see Supplementary Information for details of the motion parameters for the rsFC analysis). To assess connectivity, we extracted correlation coefficients between MFG and dACC seeds (Fig. 1b) at baseline and week 4 of the intervention. 21 of the 29 participants (72%) showed an increase in rsFC between seeded regions of the CCN after 4 weeks of EVO.

#### Cognitive control performance (AID/CPT)

**Fig. 2:**
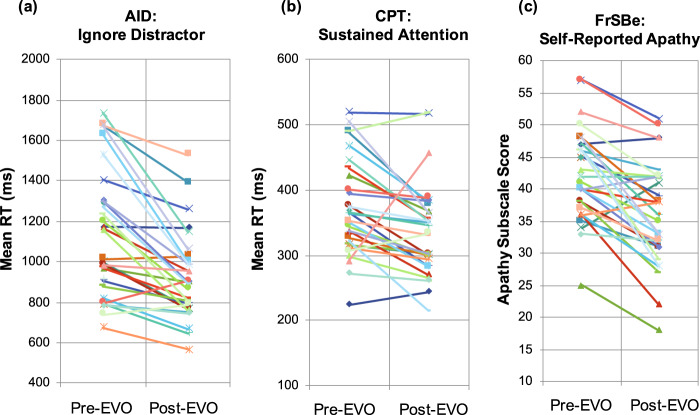
Improvement in cognitive control performance and self-reported cognitive control symptoms following 4 weeks of the EVO intervention. **a** Change in mean response time (RT) from pre- to post-EVO on the Ignore Distractor condition of the AID task, a measure of working memory. (Pre-EVO: *M* = 1141.50 ms (321.23); post-EVO: *M* = 920.98 ms (214.39); pre vs. Post: t(29) = 5.92, *p* < 0.001). **b** Change in mean RT from pre- to post-EVO on the Sustained Attention condition of the CPT. (Pre-EVO: *M* = 1069.38 ms (261.25); Post-EVO: *M* = 886.67 ms (204.19); pre vs. Post: t(29) = 5.00, *p* < 0.001). **c** Change in score from pre- to post-EVO on the Apathy Subscale score of the FrSBe self-report scale. (Pre-EVO: M = 42.30 (7.13); Post-EVO: *M* = 36.03 (8.01); pre vs. post: t(29) = 5.82, *p* < 0.001).

Twenty-three of the 34 participants (68%) showed an improvement ≥0.5 on our composite Z-score of CCN performance (on the AID and/or the CPT task) after 4 weeks of video game use. Participants were significantly faster from pre- to post-intervention on the ignore distractor condition of the AID and the sustained attention condition of the CPT (Fig. 2a, b).

#### Self-reported cognitive control symptoms (FrSBe)

Twenty-three of the 34 participants (68%) showed an improvement ≥0.5 on our composite *Z*-score of self-reported CCN symptoms (on the AID and/or the CPT task) after 4 weeks of EVO. Self-reported apathy was significantly reduced pre- to post-intervention (Fig. 2c).

For exploratory subgroup analyses examining relationships of gender, age, and duration of gameplay to target engagement, see Supplementary Table 1.

### Secondary analyses

#### Whole-brain fMRI analysis

*Task-based activation following 4 weeks of therapeutic video game use*

**Fig. 3:**
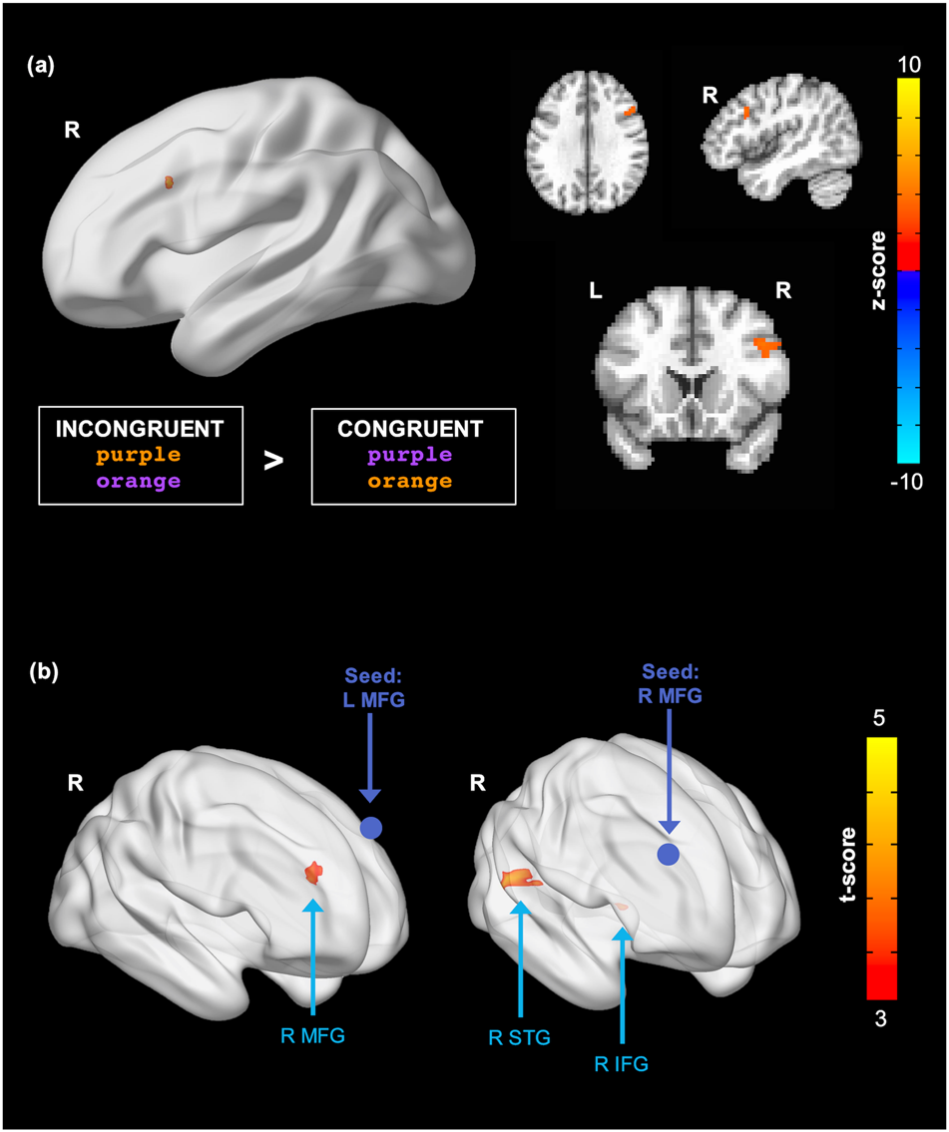
Exploratory whole-brain analysis of changes in task-based activation and resting-state functional connectivity (rsFC) following 4 weeks of the intervention. **a** Greater activation at post-EVO in the right middle frontal gyrus (MFG) during the Stroop/Flanker task for incongruent trials relative to congruent trials; voxel threshold: *p* < 0.005, cluster threshold: *p* < 0.07. **b** Increased functional connectivity from pre- to post-EVO from the left MFG seed to the right MFG and the right MFG seed to the right superior temporal gyrus (STG) and right inferior frontal gyrus (IFG); voxel threshold: *p* < 0.001, cluster threshold: *p* < 0.05.

To explore the pattern of whole-brain activation following use of the intervention, we assessed activation of incongruent relative to congruent Stroop/Flanker trials A group contrast map showing all incongruent trials minus all congruent (collapsed across Flanker similarity) trials at week 4 revealed a positive cluster (more activation for incongruent than for congruent trials) surviving cluster correction, located in the right MFG (cluster size = 39 voxels; Peak coordinates: 50, 21, 30.5; Fig. 3a). Participants also demonstrated improved performance on the Stroop/Flanker task after 4 weeks of EVO (see Supplementary Fig. 3).

*Resting-state connectivity following 4 weeks of therapeutic video game use*

Increased rsFC following EVO was observed between the left MFG seed and a cluster located contralaterally in the right MFG (cluster size = 50 voxels; peak MNI coordinates: 30, 48, 27; peak intensity = 5.4174). Increased rsFC following EVO was also observed between the right MFG seed and two clusters: one located in the right inferior frontal gyrus (IFG) (cluster size = 43 voxels; peak MNI coordinates: 42, 24, 9; peak intensity = 4.4314) and one located in the right superior temporal gyrus (STG) (cluster size = 95 voxels; peak MNI coordinates: 54, −36, 15; peak intensity = 5.0816). Significant clusters are displayed in Fig. 3b.

### Improvement in CCN circuitry with therapeutic video game use is associated with post-intervention cognitive control performance and self-reported apathy

**Fig. 4:**
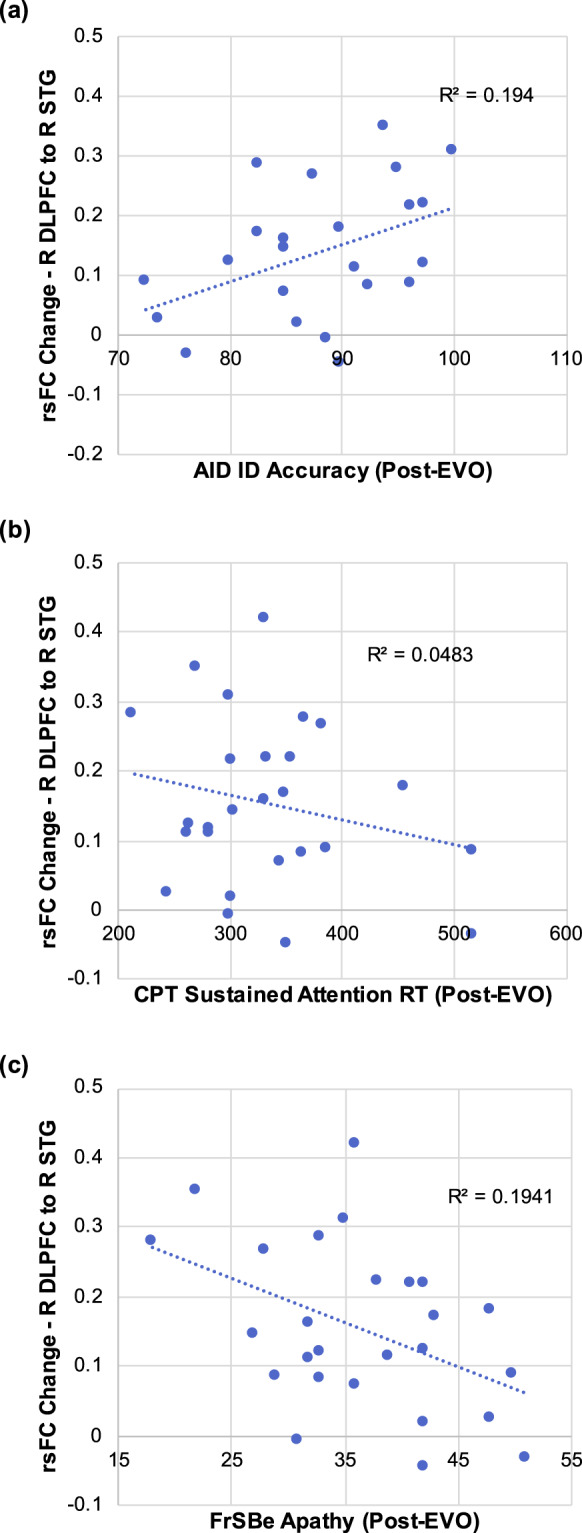
Association between pre- to post-EVO change in CCN rsFC is and cognitive control performance and self-reported symptoms post-EVO. **a** Change in rsFC between the R MFG and R STG is significantly correlated with accuracy on the “Ignore distractor” condition of the AID task post-EVO. **b** Change in rsFC between the R MFG and R STG is significantly not correlated with performance on the “Sustained Attention” condition of the CPT post-EVO. **c** Change in rsFC between the R MFG and R STG is significantly correlated with post-intervention scores on the Apathy Subscale score of the FrSBe self-report scale.

We observed change in rsFC of the CCN following 4 weeks of video gameplay was associated with post-intervention scores on behavioral and self-report measures of CCN target engagement. Specifically, pre- to post-EVO change in rsFC between R MFG and R STG was associated with greater working memory ability (measured by AID performance in the “ignore distractor” condition) post-intervention (*r*(23) = 0.44, *p* = 0.03; Fig. 4a), but was not significantly correlated with sustained attention (measured by CPT performance) post-intervention (*r*(24) = −0.22, *p* = 0.28; Fig. 4b). Pre- to post-EVO change in rsFC between R MFG and R STG was associated with reduced self-reported apathy (measured by FrSBe self-report score) post-intervention (*r*(24) = −0.44, *p* = 0.02; Fig. 4c).

### Exploratory analysis

*Improvement in self-reported mood symptoms over time*: we observed a significant improvement in depression severity over time, as measured by PHQ-9 score (by repeated-measures ANOVA, F[4,25] = 16.5, *p* < 0.001; Supplementary Fig. 4). Among 32 individuals with baseline and week 4 PHQ-9, 13 (41%) met the criteria for treatment response and 7 (22%) for remission.

## Discussion

In this investigation of a neuroscience-informed therapeutic video game-like intervention for MDD, we confirmed the engagement of CCN using brain-based measures (task-based fMRI, resting-state fMRI), sustained attention or working memory performance (CPT, AID), and self-reported behavioral symptoms of cognitive control disturbance (Apathy subscale of the FrsBE). Specifically, we found that a digital therapeutic strategy designed to improve cognitive control abilities (i.e., multitasking) improved brain-based focal activation and rsFC functional connectivity on fMRI of the CCN in more than 70% of our sample of middle-aged and older adults with MDD and cognitive control dysfunction. In parallel, we identified significant improvements in performance and self-reported CCN functions in 68% of participants. Taken together, these convergent results demonstrate target engagement of the CCN.

These findings extend our prior proof-of-concept randomized controlled trial conducted in an elderly sample of individuals suffering from a major depressive episode. That trial demonstrated similar antidepressant efficacy for the game intervention and PST, but only the former was associated with improvement in cognitive control performance^13^. However, that study could not address the mechanism of action. Here, we demonstrate that improvement in executive functions and depression may be the results of a change in activation and functional connectivity of CCN, validating this novel treatment target.

As in the prior study, our exploratory analyses found this gamified intervention to have a significant impact on mood after 4 weeks of treatment. More than half of those treated met the criteria for symptomatic response, and one quarter for remission. Notably, this is a sample characterized with risk factors for poor response to antidepressant treatment^7,36–40^, in which response rates after 8 weeks of antidepressant medication treatment have been reported to be between 25% and 60%^7,40^.

Our study adds to a growing literature indicating that gamified interventions may impact depressive symptoms^41–44^. Recent meta-analyses of prior cognitive training interventions showed a small to moderate effect size for symptom improvement (0.43) in depression^45^. The majority of these trials have been relatively small and, with few exceptions^42,43^, have not directly interrogated the underlying neural mechanisms tied to improved mood-based symptoms. In this study, the intervention was designed to selectively improve CCN functions that are both susceptible to effects of normal aging and predictive of poor antidepressant response in older individuals with MDD. The current findings, in conjunction with our previous results, suggest that the engagement of the CCN through repeated cognitive stimulation may be a central mechanism for rescuing dysfunctional brain networks that contribute to both the mood and cognitive symptoms in many suffering from MDD.

We note several caveats. Although we demonstrate significant changes in CCN functions using converging methodologies, without a control group we cannot state with confidence that these changes are attributable to the intervention until it is compared to an active, equally engaging control. Further, the modest sample size requires replication of the target engagement findings. Likewise, changes in mood could be a function of regularly engaging in an entertaining activity or receiving weekly visits with a care manager. Participants completed the AID and CPT tasks at baseline and week 4 of the study so practice effects may account for some of the improvement in performance following treatment. We also cannot assess the specificity of effect—i.e., whether the similar benefit would be observed in individuals with MDD but without executive dysfunction. It is possible that only those with dysfunction in the CCN will benefit from an intervention designed to target this deficit^46^, whereas this approach may not be effective for individuals whose depression may be mediated by deficits in other brain networks^47^. Preliminary analyses suggest age and gender may be potential moderators of target engagement. However, In light of the modest sample size and risk for type 1 and type 2 error, such associations require confirmation in larger studies.

## Conclusions

This study suggests that a remotely deployed neuroscience-informed video game-like intervention improves both CCN functions and mood in middle-aged and older adults with MDD. This phase of a two-phase clinical trial focused on the ability of a video game-like intervention designed to improve multitasking to engage the CCN. The next steps require confirmation of engagement of the CCN target in a randomized trial that includes an expectancy-matched video game control played at the same frequency and duration as the experimental intervention. Subsequent investigation in a larger cohort will also be required to identify whether particular patient subgroups benefit differentially in terms of both CCN function and mood symptoms. Nevertheless, this study underscores the importance of the CCN as a target for interventions aimed at the treatment of MDD, addressing symptoms that are frequently resistant to traditional antidepressant treatments in mid-to-late adulthood.

## Supplementary information

Supplemental Material
