# Supplementary material for: A digital intervention targeting cognitive control network dysfunction in middle age and older adults with major depression
Source: Transl Psychiatry. 2021 May 4;11:269. doi: 10.1038/s41398-021-01386-8 (PMC8096948; doi:10.1038/s41398-021-01386-8)
Supplement: Supplementary file 1 — Supplemental Material [file 41398_2021_1386_MOESM1_ESM.docx]

**Supplementary Information**


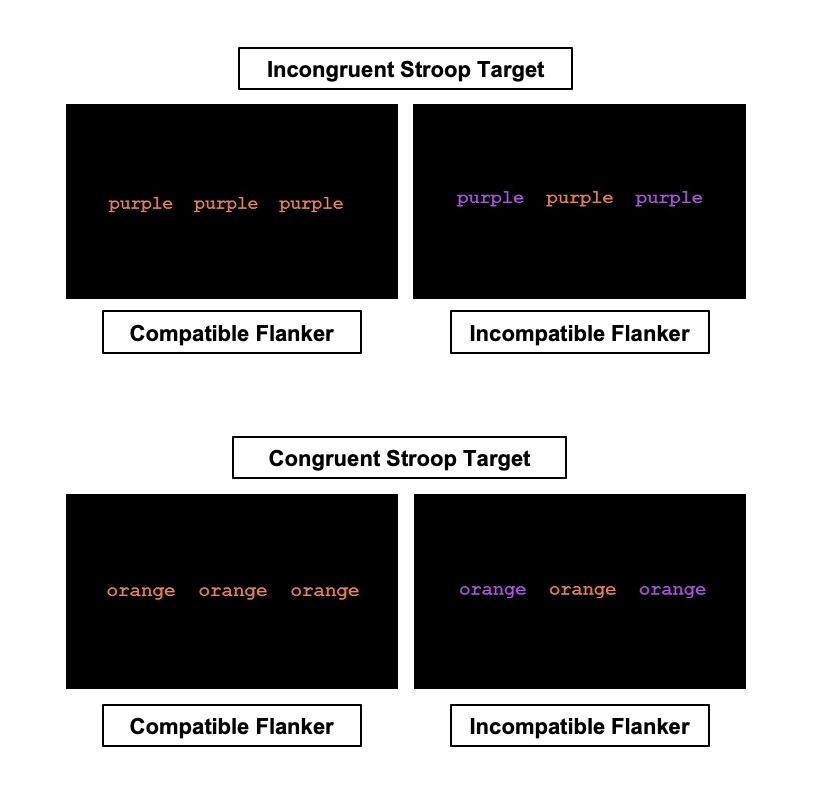


***Supplementary Figure 1:*** *Stroop/Flanker Task.* The Stroop/Flanker task measures multiple aspects of cognitive control, including the updating and maintenance of representations of the environment, goal-directed behaviors, response selection, and inhibition of attention towards distracting, irrelevant information. In this task, participants viewed three color words on the screen: the central Stroop target word and two surrounding Flanker words. Participants responded to the ink color of the central target word with a button press, while ignoring multiple sources of cognitive interference: what the word says and the surrounding distractor word. The Stroop targets were congruent (e.g., “purple” in purple ink) or incongruent (e.g., “purple” in orange ink). The distractors will be compatible (same ink color as the target) or incompatible (different ink color than the target). The combined use of the Stroop and Flanker paradigms in a single cognitive control task was designed to be more taxing on the CCN, with successful performance depending upon monitoring and resolution of both within-stimulus and between-stimulus attributes. Participants completed two runs of 96 trials of the Stroop/Flanker task in the scanner. The task was displayed using e-Prime stimulus presentation software (Psychology Software Tools).^S7^ Each trial was displayed until the participant made a button press response or for a maximum of 2000ms. Behavioral response times and neural activation were analyzed as a function of target congruency and distractor compatibility, with the primary contrast of interest being activation and of the CCN in response to incongruent vs. congruent stimuli.

***Supplementary Figure 2.*** *Participant flow for sample included in analysis.* Individuals were screened before enrollment for a diagnosis of Major Depressive Disorder without psychotic features and a deficit in cognitive control network (CCN) function*.* Of the 41 participants who met criteria and enrolled, 34 participants completed the study and were included in the final analysis. The full sample of 34 participants were included in the analysis of task-based MRI activation, performance data, and self-reported symptoms of CCN function; 29 participants were included in the functional connectivity analysis.

***Supplementary Methods: MRI Image Acquisition & Preprocessing***

*Image Acquisition:*

MRI data were acquired on a 3T Siemens TIM Trio scanner (Erlangen, Germany) at the Center for Biomedical Imaging and Neuromodulation (C-BIN) of the Nathan Kline Institute for Psychiatric Research and a 3T Philips scanner at the Diagnostic Imaging Sciences Center (DISC) of the University of Washington. Structural and functional images were acquired with a 32-channel head coil. Resting-state data and task-based Stroop/Flanker data were whole-brain T2-weighted echo planar images (EPI) with BOLD contrast (218 volumes per run; 34 slices with no gap; interleaved acquisition; TR = 2400ms; TE = 30ms; voxel size = 2.5mm x 2.5mm x 3.5mm; FoV = 240mm; flip angle = 80°). We also acquired a high-resolution T1-weighted MPRAGE scan for registration of functional data (192 slices with no gap; interleaved acquisition; TR = 2500ms; TE = 3.5ms; voxel size = 1mm isovoxel; FoV = 256mm; flip angle = 8°). The resting-state scan and the 2 runs of the Stroop/Flanker task lasted approximately 7 minutes and 30 seconds each.

*Task-Based Preprocessing:*

To examine CCN activation during the Stroop/Flanker task, we extracted baseline and week 4 response times for each participant for incongruent and congruent Stroop/Flanker trials. Stimulation timing text files were created for further use in general linear modeling (GLM) analyses. The preprocessing of the task-based data was performed with the Analysis of Functional Images (AFNI version 18.2.15; https://afni.nimh.nih.gov/).^S1^ Preprocessing inputs were the task-based EPI datasets for run 1 and 2 for each participant, and these images were co-registered to the T1-weighted MPRAGE. We performed slice-timing correction, skull stripping, anatomical warping to standard space, blurring with s FWHM of 4 mm, and scaling of each voxel times series to a mean of 100. TRs having an estimated motion greater than 0.9 mm were censored. 3dDeconvolve was used to detrend, regress motion parameters and task-specific events, as well as to set the general linear test. The default basis regression function GAM was used in 3dDeconvolve. Participants who had a whole-brain signal-to-noise-ratio (TSNR average) < 100 at either baseline or follow-up were excluded from further task-based analysis. The incongruent vs. congruent contrast was performed using the AFNI command 3dttest++, restricted to an ROI-mask of the cognitive-control network (CCN). The CCN mask was created combining anatomical regions of the Harvard cortical atlas (bilateral anterior cingulate, superior, middle and inferior frontal gyri, frontal pole, superior parietal lobe, supramarginal and angular gyri). AFNI ClustSim was used to calculate alpha levels for cluster correction. We extracted Beta values from significant clusters, statistically thresholded with a voxel level p-value < 0.005 and a cluster level p-value < 0.07.

*Resting-State Preprocessing:*

The preprocessing of resting state data was performed using Data Processing Assistant for Resting-State fMRI (DPARSF) (4.0 Advanced Edition; Yan 2010), a software plug-in within DPABI (http://rfmri.org/dpabi),^S2^ which is part of SPM.^S3^ Resting-state images were cropped, brain extracted, and co-registered to the T1-weighted MPRAGE. Segmentation into grey matter (GM), white matter (WM), and cerebrospinal fluid (CSF) was performed based on SPM priors. Nuisance regression was performed with WM, CSF, and Friston 24 motion parameters as covariates. We applied CompCor (Behzadi et al. 2007), in place of Global signal regression (GSR), to minimize the impact of signal related to physiological noise. Images were normalized using EPI templates and smoothed with a Gaussian kernel of 6 mm FWHM. The data were filtered within a range of 0.01-0.1 Hz. Lastly, to reduce physiologic sources of head displacement, all functional images were scrubbed with removal of bad time-points (TR with estimated motion greater than 0.5mm).^S4^ We excluded participants from further rsFC analysis if remaining data after scrubbing were ≤ 105 volumes or if mean framewise displacement (FD) was > 2 SDs above the group mean. Whole brain r-to-z functional connectivity maps were (zFC maps) calculated for each participant seeds with a 4mm radius placed bilaterally in the middle frontal gyrus (MFG; MNI coordinates -36/36, 28, 34) and the dorsal anterior cingulate cortex (dACC; MNI coordinates -4/4, 30, 22). We ran paired t-tests to calculate statistical maps for each seed. Resulting functional maps were thresholded for cluster-level correction with a Gaussian Random Field (GRF). We statistically thresholded the zFC maps with a voxel level p-value < 0.001 and a cluster p-value < 0.05. Correlation coefficients were extracted from significant clusters from each zFC map.

The resting-state preprocessing parameters, particularly the motion threshold of 0.5mm, were selected based on the work of Power et al., 2017.^S4^ This analysis approach has found to be effective in censoring high motion volumes from resting-state MRI data and improving data quality. Slightly different preprocessing parameters, including the less conservative motion and statistical thresholds, were used for the task-based analysis. This approach is consistent with findings indicating that distinct processing strategies may be appropriate for resting-state vs. task-based data, and less conservative motion censoring may improve the modeling of task events and increase statistical power.^S5,S6^

***Supplementary Figure 3:*** *Improvement in Stroop/Flanker Performance Following EVO.* Participants were significantly faster in all trial conditions pre- to post-treatment, including incongruent Stroop trials (Pre-EVO: M = 826.68 (136.97); Post-EVO: M = 772.23 (122.00); Pre vs. Post: t(33) = 3.42, p = 0.002) and congruent Stroop trials (Pre-EVO: M = 736.78 (104.28); Post-EVO: M = 697.21 (104.81); Pre vs. Post: t(33) = 2.91, p = 0.006), collapsed across Flanker similarity. The effect size for improvement on task performance was greater for the incongruent trials (*Cohen’s d* = 0.42) than the congruent trials (*Cohen’s d* = 0.37), indicating that treatment with EVO had a greater effect on performance conditions with a greater cognitive load.

******

***Supplementary Figure 4:***  *Overall improvement in self-reported mood symptoms during 4 weeks of EVO treatment.* Weekly depression severity measured by PHQ-9 scale scores. Bonferroni-corrected p-value: * p < 0.05; ** p < 0.001

**Table 1:** Proportion of participants meeting target engagement goal for each level of analysis of cognitive control function. The successful target engagement goal was defined as 66% of participants with significant improvement (z-score ≥ 0.5) in CCN measures (performance, self-report, circuitry) following 4 weeks of EVO.

Subgroup analysis for gender, age (median split; *Younger* Age Group: < 63 years old; *Older* Age Group: ≥ 63 years old), and gameplay (median split of total number of sessions played during treatment; Less Gameplay Group = < 118 sessions; More Gameplay Group: ≥ 118 sessions; range = 74-164 sessions) are reported, and group differences are assessed with chi-squared tests.

| **Level of Analysis** | **Overall:**  **N (%)** | **Differences by**  **Gender: N (%)** | **x^2^**  **(p-value)** | **Differences by**  **Age: N (%)** | **x^2^**  **(p-value)** | **Differences by Gameplay: N (%)** | **x^2^**  **(p-value)** |
| --- | --- | --- | --- | --- | --- | --- | --- |
| Cognitive Control Performance: AID/CPT | 23/34 (68%) | Male: 7/10 (70%)  Female: 16/24 (67%) | 0.07  (0.80) | Younger: 10/16 (63%)  Older: 13/18 (72%) | 0.60  (0.44) | Less: 13/17 (76%)  More: 10/17 (59%) | 2.14  (0.14) |
| Self-Reported Cognitive Control Symptoms: FrSBe | 23/34 (68%) | Male: 7/10 (70%)  Female: 16/24 (67%) | 0.07  (0.80) | Younger: 10/16 (63%)  Older: 13/18 (72%) | 0.60  (0.44) | Less: 12/17 (71%)  More: 11/17 (65%) | 0.27  (0.61) |
| CCN Circuitry (Task-Based Activation) | 25/34 (74%) | Male: 5/10 (50%)  Female: 20/24 (83%) | 8.19  (0.004)* | Younger: 13/16 (81%)  Older: 12/18 (67%) | 1.32  (0.25) | Less: 14/17(82%)  More: 11/17 (65%) | 1.97  (0.16) |
| CCN Circuitry (rsFC) | 21/29 (72%) | Male: 5/8 (63%)  Female: 16/21 (76%) | 1.22  (0.27) | Younger: 9/15 (60%)  Older: 12/14 (86%) | 4.63  (0.03)* | Less: 12/15 (80%)  More: 9/14 (64%) | 1.78  (0.18) |

*Note: CPT = Continuous Performance Task; FrSBe = Frontal Systems Behavior Scale; CCN = Cognitive Control Network; rsFC = Resting-State Functional Connectivity*

** Group difference in percentage of target engagement is significant at the p < 0.05 level.*

This preliminary analysis of differences in CCN target engagement as a function of gender, age, and gameplay suggested select influences of gender and age on target engagement. That is women showed a higher rate of target engagement in task-based CCN activation whereas older participants showing greater frequency of target engagement in resting state functional connectivity of the CCN. Given the modest sample size, we will examine these *ad hoc* analyses in the next phase of this study in a larger sample that will allow for the confirmation of the potential moderating effects of gender and age. Target engagement did not differ as a function of number of sessions of gameplay.

***Supplementary References***

*S1.* Cox, RW. AFNI: software for analysis and visualization of functional magnetic resonance neuroimages. Computers and Biomedical Research, 1996; 29(3), 162-173.

S2. Yan CG, Wang XD, Zuo XN, Zang YF. DPABI: data processing & analysis for (resting-state) brain imaging. Neuroinformatics. 2016;14(3):339-51.

S3. Penny WD, Friston KJ, Ashburner JT, Kiebel SJ, Nichols TE, editors. Statistical parametric mapping: the analysis of functional brain images. Elsevier; 2011 Apr 28.

S4. Power JD, Plitt M, Kundu P, Bandettini PA, Martin A. Temporal interpolation alters motion in fMRI scans: Magnitudes and consequences for artifact detection. PloS one. 2017 Sep 7;12(9):e0182939.

S5. Mayer AR, Ling JM, Dodd AB, Shaff NA, Wertz CJ, Hanlon FM. A comparison of denoising pipelines in high temporal resolution task‐based functional magnetic resonance imaging data. Human brain mapping. 2019;40(13):3843-59.

S6. Siegel JS, Power JD, Dubis JW, Vogel AC, Church JA, Schlaggar BL, Petersen SE. Statistical improvements in functional magnetic resonance imaging analyses produced by censoring high‐motion data points. Human brain mapping. 2014;35(5):1981-96.

S7. Schneider, W., Eschman, A., and Zuccolotto, A. E-Prime User’s Guide. 2012; Pittsburgh: Psychology Software Tools, Inc.
